# Getting a Grip on Complexes

**DOI:** 10.2174/138920209789503923

**Published:** 2009-12

**Authors:** Yan Nie, Cristina Viola, Christoph Bieniossek, Simon Trowitzsch, Lakshmi Sumitra Vijay-achandran, Maxime Chaillet, Frederic Garzoni, Imre Berger

**Affiliations:** European Molecular Biology Laboratory (EMBL), Grenoble Outstation and Unit of Virus Host-Cell Interactions (UVHCI), UJF-EMBL-CNRS, UMR 5233, 6 rue Jules Horowitz, 38042 Grenoble CEDEX 9, France

**Keywords:** Proteome, interactome, multiprotein assemblies, structural genomics, robotics, multigene expression, multiBac, BEVS, ACEMBL, complexomics.

## Abstract

We are witnessing tremendous advances in our understanding of the organization of life. Complete genomes are being deciphered with ever increasing speed and accuracy, thereby setting the stage for addressing the entire gene product repertoire of cells, towards understanding whole biological systems. Advances in bioinformatics and mass spectrometric techniques have revealed the multitude of interactions present in the proteome. Multiprotein complexes are emerging as a paramount cornerstone of biological activity, as many proteins appear to participate, stably or transiently, in large multisubunit assemblies. Analysis of the architecture of these assemblies and their manifold interactions is imperative for understanding their function at the molecular level. Structural genomics efforts have fostered the development of many technologies towards achieving the throughput required for studying system-wide single proteins and small interaction motifs at high resolution. The present shift in focus towards large multiprotein complexes, in particular in eukaryotes, now calls for a likewise concerted effort to develop and provide new technologies that are urgently required to produce in quality and quantity the plethora of multiprotein assemblies that form the complexome, and to routinely study their structure and function at the molecular level. Current efforts towards this objective are summarized and reviewed in this contribution.

## INTRODUCTION

Protein-protein interactions (PPIs) are intrinsic to virtually every essential process in the cell. Deciphering PPIs is imperative for understanding the underlying biological mechanisms of living systems. Cellular activities that govern health and disease, such as DNA replication, transcription, splicing, translation, secretion, cell cycle control, signal transduction and intermediary metabolism are controlled by PPIs [1-5]. New developments in sequencing technology in combination with advances in affinity purification techniques and automation are presenting researchers with the opportunity to study the proteome of various organisms at an ever increasing pace. Genome-wide protein-protein interaction studies involving affinity chromatography and mass spectrometry (MS) analyses of systematically tagged open reading frames (ORFs) have been developed and implemented, aided by powerful bioinformatics approaches, to address the entirety of PPIs in cells.

To date, many thousands of PPIs are known, however, the precise molecular details are available for only a small fraction of these interactions. Structure elucidation can ultimately turn abstract system representations into models that more accurately reflect biological reality. The utility of structural biology is to understand the mechanisms governing biological interactions in living systems for designing strategies to modulate, and interfere with these interactions. However, the large and increasing body of data describing PPIs on a genome-wide scale, and the pace at which it is amassed, is currently at a pronounced disparity with the rate at which the structure and function of representative protein complexes that comprise the identified interactions, are described at the molecular level. Despite considerable advances in contemporary structure determination techniques and significant efforts by structural genomics consortia to streamline the process leading to high-resolution structures, many bottlenecks in the structure determination pipeline remain.

Protein complexes are often found in scarce amounts in their endogenous host and remain difficult to isolate in the quantity and quality required for detailed functional and structural analysis. This is often the case already for electron microscopy experiments, although the requirements of this technique in terms of sample quantity are typically less imposing as compared to studies for example by X-ray crystallography or by nuclear magnetic resonance (NMR) spectroscopy. The latter two are the currently most powerful and widely used techniques for providing high-resolution structural information. Multiplexed overexpression experiments by using advanced recombinant production technologies could be instrumental not only for overcoming the sample production bottleneck, but also for compellingly validating proposed interactions in a heterologous setup. Streamlined high-throughput technologies for production of multisubunit protein complexes, however, have been utterly lacking to date. New developments are required to rapidly and reproducibly construct large protein complexes and variations thereof at the rate that they are conceptualized from genome-wide studies.

## DECIPHERING THE INTERACTOME

In recent years, new and powerful methods have been developed which allow complex cellular protein-protein interaction networks to be mapped (Fig. (**1**)). Such techniques have produced a wealth of data and have given rise to a new sphere of research designated “interactomics”. The term “interactome” is used to describe all known interactions present in the cellular gene product repertoire [6].

### Purification from Native Source

A celebrated development in high-throughput identification of protein complexes is the tandem affinity purification (TAP) method [7]. In this approach endogenously tagged proteins of interest are produced which are used as bait to fish out interacting partners. The original TAP tag comprises two affinity tags: the Z-domain of protein A, which binds to immunoglobulin G (IgG), and calmodulin-binding peptide (CBP), which binds to calmodulin. These two tags are separated by the highly specific tobacco etch virus (TEV) protease site. TAP tagging involves a relatively mild extraction procedure in which protein complexes are purified *via* a two-step process that yields intact protein complexes composed of the tagged bait and any associated partners. This method is particularly useful for detecting stable complexes; more transient complexes are not observed, as they tend to dissociate during purification. Two major proteome-wide studies in *S. cerevisiae* using the TAP method have revealed many previously unknown protein interactions and pathway associations [8, 9]. In one study, Gavin *et al*. TAP-tagged 6406 ORFs from the *S. cerevisiae* genome which enabled the purification of 1993 tagged proteins and the identification of 491 protein complexes [8]. In an independent study, Krogan *et al*. TAP-tagged 4562 ORFs from the yeast proteome. 2357 of these TAP-tagged proteins were purified revealing 547 complexes as well as 429 interactions between complexes [9]. In both of these extensive studies affinity tags were introduced into the 3’ ends of target ORFs in the yeast chromosome by homologous recombination. Data generated from these surveys correlated well with known protein complexes formerly discovered and studied by conventional means. More notably, new interaction partners of well-known complexes were identified, as well as entirely novel complexes and associations.

Methods to optimize the TAP tagging strategy are under way in an effort to obtain larger quantities of tagged protein assemblies. One of the challenges of the TAP method is to gain insight into the more fleeting interactions present in a protein complex. Herzberg *et al.* have developed a Strep-protein interaction experiment (SPINE) that deals with the inherent false positives otherwise found in TAP tagging experiments [10]. By replacing the TAP tag with a strongly interacting variant of Streptavidin called Strep-tactin and employing a reversible cross-linking reagent, Herzberg *et al*. were able to get an *in vivo* snap-shot of bait interactors in *B. subtilis* in a single affinity purification step.

In the years since the pioneering initial glimpses into the yeast interactome, subsequent affinity purification studies have sought to shed light on the interactomes of multicellular organism. Multicellular organisms are generally less amenable to TAP-tagging approaches due to the challenge of using homologous recombination to insert affinity tags and the difficulties in retrieving sufficient quantities of purified material. Nevertheless, Cheeseman *et al*. described a procedure using the TAP tagging principle to purify protein complexes from *C. elegans* strains and cultivated HeLa cells [11]. By modifying the TAP tag to include green fluorescent protein (GFP) followed by the Z-domain of protein G instead of protein A, and by replacing the CBP-tag with streptavidin peptide, this study revealed intact complexes involved in *C. elegans* kinetochore formation.

Furthermore, Burckstummer *et al*. overcame the problem of low protein yields in TAP tagging experiments in mammalian systems by likewise altering the composition of the TAP tag [12]. They also replaced the IgG peptides of Protein A with those of Protein G and the CBP peptide with streptavidin peptide. Using IKKγ with this modified TAP tag as bait resulted in a ten-fold increase not only in the amount of bait but also of its interacting partner, IKKα. These advancements in affinity purification techniques promise to allow future interactome maps of cultivated human cell lines to be determined, as well as maps of other cell types that are inherently more difficult to cultivate in large quantities, such as neuronal cells and immune cells. By tweaking certain aspects of existing purification strategies, such as modifying the original TAP tag itself, high-throughput interactome maps are moving into the realm of mammalian systems.

An interesting approach called BAC TransgeneOmics was recently described as a tool for studying protein-protein and protein-DNA interactions in addition to protein localization [13]. BAC TansgeneOmics describes a method by which all known proteins within a proteome of a given organism are tagged on a genome-wide scale. Using this recombinantly tagged genome to create a bacterial artificial chromosome (BAC) library ensures the presence of native regulatory regions around the target gene. BACs containing the recombinantly tagged genes of interest are then sequentially transfected and expressed in mammalian cells. The tags consist of a combination of fluorescent proteins and peptides for affinity purification and reporting on factors such as *in vivo* protein localization and endogenous protein interactions.

### Interaction Analysis by Yeast Two-Hybrid Screens

Another powerful method for generating interactome maps in a high-throughput manner is the yeast two-hybrid (Y2H) approach [14]. Interactome-wide binary interaction maps resulting from Y2H screens are generally regarded as low-coverage studies, noisy and containing a high likelihood of false positives. In an attempt to systematically map interactome networks from Y2H screens, Venkatesan *et al.* estimate that only 8% of the full human interactome has been covered by Y2H screens [15]. However, these surveys continue to provide a useful concomitant view of the whole interactome when considered alongside other affinity purification/MS-based techniques [5]. Y2H screens report on whether or not two proteins interact by fusing to a target protein the DNA binding domain (DBD) of a transcription factor while potential binding partners are fused to an activation domain. Any interaction between the two target proteins leads to the expression of a reporter gene [16]. There are three commonly used high-throughput Y2H screening approaches: (1) the yeast mating approach in which haploid DBD strains and strains with the activation domains undergo mating and selection for reporter expression; (2) the matrix approach, where DBD strains can be mated with an array of strains containing activation domains; and (3) the library approach, which involves the mating of individual DBD strains with a library of activation domain strains that represents a cDNA library of a given target organism [5]. The latter method is the most efficient for high-throughput studies, however, the sampling efficiency of individual DBD strains with entire cDNA libraries is greatly reduced.

While the Y2H strategy has the capacity to meet the demands of high-throughput interactome mapping, this approach cannot currently compete with affinity based methods in terms of genome coverage. Nonetheless, Y2H surveys have realized a rich source of high-quality binary interaction maps from a wide range of organisms, including viruses, bacteria [17], *S. cerevisiae* [14, 18, 19], *D. melanogaster* [2], *C. elegans* [20-22] and humans [4, 23, 24]. It is also important to note that while Y2H screens are critisized for inherent problems concerning the overexpression of homologous genes, the post-translational modification machinery and a bias towards interactions that occur in the nucleus, this approach can examine a different subspace of the protein interaction world to that sampled by affinity/MS methods. Together, both sources of interactome mapping provide a more comprehensive outlook of the whole interactome.

Two valuable high-throughput Y2H human PPI maps were generated by Stelzl *et al*. [24] and Rual *et al*. [4]. These independent studies both utlized the matrix approach to achieve greatest possible coverage of the human genome and between them identified approximately 6000 binary protein interactions. In the Stelzl study, where 4456 baits and 5632 preys were screened, 195 disease related genes were found to interact with previously unidentified partners. Furthermore, 342 uncharacterized proteins were assigned new putative roles after being found to interact with a protein of known function. In total, new functions were assigned to hundreds of different proteins. In a comparable effort, Rual and colleagues looked for binary interactions between approximately 8100 ORF’s and detected approximately 2800 protein associations. These interactions were then correlated with independant co-affinity purifications which revealed an overlap of approximately 78%. Despite the impact these Y2H screens have made in the field of interactomics, further developments are still required before they reach the coverage achieved by affinity methods. The impact of these studies will surely propel the current technology in Y2H to new heights.

In a recent high-quality yeast binary protein interaction study, Yu *et al.* have attempted to deal with a long standing criticism that Y2H screens are biased towards interactions that occur within the nucleus [25]. To counter this concern, Yu *et al.* performed a Y2H screen in parallel with a yellow flourescent protein complementation assay (PCA) in which the traditional bait and prey peptides are replaced with non-flourescing halves of yellow fluorescent protein (YFP). Once the interacting partners are in close proximity, the fluorescent properties of YFP are reconstituted and thereby create a useful marker that is not limited to reporting on interactions that occur within the nucleus. Using their dual method, Yu *et al.* were able to validate their own results, which showed a greater degree of correlation than that shown between the Gavin and Krogan TAP studies. Y2H screens are certainly becoming a valuable tool for studying genome-wide protein ineractions and will likely continue to make major contributions to the field of interactomics.

### Computational Approaches 

Results from high-throughput interactome studies are being tabulated with increasing clarity. These efforts are resulting in unprecedented amounts of potentially useful data for molecular and structural biologists. On the bioinformatics side, the major hurdles in analyzing high-throughput interactome data sets include managing databases, creating useful clustering algorithms to glean valuable information about protein interactions, and using the resulting clustering to make predictions about biological systems. Results from combined genome-wide interaction studies may contain only partially overlapping datasets, false positives (interactions that should not normally occur in a cell) and false negatives (limited or biased coverage that excludes a true interaction). Such issues hamper a comprehensive portrayal of protein networking [26]. Today’s bioinformatician faces many challenges in the emerging field of interactomics. What follows is an overview of what challenges are being faced currently and those that are on the horizon that will undoubtedly continue to be a boon for structural biologists in search of complex three dimensional (3-D) structures.

Considering that each genome-wide interactome study generates characteristic data and that each existing repository uses characteristic file formats for storing data, the challenge of creating a consolidated resource for a transparent flow of data between datasets is startling. The Molecular Interactions (MI) group of the Proteomics Standards Initiative (PSI) has created an international standard for representing protein interaction data by consolidating existing interactome data sets from individually curated databases to create the International Molecular Interaction Exchange consortium (IMEx) [27]. The consortium, to date, includes the following databases: DIP (http://dip.doe-mbi.ucla.edu), IntAct (http://www.ebi.ac.uk/intact), MINT (http://mint.bio.uniroma2.it/mint), MPact (http://mips.gsf.de/genre/proj/mpact), MatrixDB (http://www.matrixdb.ibcp.fr), BioGRID (http://www.thebiogrid.org), MPIDB (http://www.jcvi.org/mpidb) and BIND (http://www.blueprint.org). Alongside IMEx is MIMIx, the minimum information required for reporting a molecular interaction experiment. MIMIx tackles the lack of community consensus on what information is required to report molecular interaction by setting up an international standard to facilitate the extraction of useable data from PPI experiments by users. Currently, data is exchanged in XML format.

A major challenge concerning interactome datasets is how to cluster the resulting interactions to accurately report on real protein complexes rather than spurious, or false positive interactions while including more transient members of protein complexes rather than only architectural ones. Based on the Gavin, Krogan and Ho studies, Hart *et al*. used an unsupervised probabilistic scoring scheme and assigned confidence scores to each interaction. This approach generated a matrix-model interpretation of the yeast interactome datasets [28]. Unsatisfied with the existing spoke model as a way of representing interactome data which only considers bait and prey interactions, Hart and colleagues devised a scoring method to hone the matrix model which additionally also takes prey/prey interactions into account, thereby including the elusive transient members of complexes without decreasing the overall accuracy of reported complexes. In doing so, it was shown that the degree of overlap between the reported datasets was considerably higher than previously thought, and that one of the major problems in previous comparisons was the inclusion of ribosomal protein interactions. Based on assessments of similarity between the above mentioned datasets and with a third yeast interactome dataset [9], Hart *et al.* suggested that these studies are approaching saturation of what can be known about the subset of the complexome of yeast grown in rich media. Recently, Krogan indicated that a rough calculation based on the overlap of the two studies suggests that approximately 80% of the interactions capable of detection in yeast by the TAP method have been detected [29].

Another consequence of the upsurge in PPI maps and genome-wide sequencing efforts is the new wealth of data that can be used by the community of scientists who model protein interactions and predict protein function from the gene sequence. With the ever increasing amounts of data about PPIs, it is possible to identify recurring ‘domain signatures’ and to correlate frequent interactions between them, the idea being that the interaction may be mediated by the signature sequence [30]. Knowledge about where an interaction might occur can also narrow down which portions of a protein sequence should be included in designing protein complex constructs [31].

### Mass Spectrometry

Mass spectrometry (MS) has emerged as an indispensable tool for studying the interactome [32, 33]. MS is now firmly established as one of the main driving forces of proteome studies, and is increasingly the method of choice for analyzing complex protein mixtures derived from entire cells. Besides protein identification, quantification and profiling, MS has had a significant impact on the analysis of protein interactions and protein complexes [32]. Combining affinity purification with MS allowed a *de novo* characterization of the composition and organization of the cellular machinery. Data derived from these methods indicated that complexes can combine transiently and differentially in a modular fashion thus enabling a diversification of the potential function of individual protein complexes [8]. MS-based interactome analysis approaches, using a variety of techniques including matrix-assisted laser desoprtion/ionization (MALDI) and liquid-chromatography coupled electro-spray ionization (LC-MS), offer several important advantages for studying protein complexes as compared to other techniques. A protein complex can be isolated directly from its cellular environment, fully processed with its full complement of modifications and directly studied by MS without the need for further manipulations [34]. MS based methods can readily detect stable interactions which constitute core architectures of protein complexes. Implementation of chemical cross-linking strategies in MS experiments further offers possibilities to detect and analyze important transient interactions [35]. A key issue is the analysis of the vast amount of data gathered in MS-based proteome and interactome analysis. Progress is being made in developing tools for analyzing MS-data based on statistical principles [36, 37].

MS experiments can likewise be used to obtain inventories of biochemically isolated organelles allowing for the characterization of sub-interactomes contained within subcellular compartments. High-resolution methods were applied for accurate protein identification and novel algorithms were developed to assign genuine components from co-purifying proteins in these experiments [38]. This holds particular promise for accessing the protein repertoire and complexome of such cellular subcompartments by high-resolution structural and functional studies.

MS based interactome wide studies are often met with skepticism concerning the reproducibility of results [39]. The Test Sample working group of the Human Proteome Organization (HUPO), who have an interest in establishing international standards for proteomics studies, attempted to address the question of irreproducibility in MS experiments. The working group provided a defined test sample containing an equimolar mixture of highly purified recombinant proteins to 27 different laboratories using high-throughput MS methods to test their ability to correctly identify the mixture [40]. The results were that, initially, only a quarter of the laboratories correctly identified the protein mixture. However, upon closer inspection of each laboratory‘s raw data, it became apparent that the peptides had in fact been identified in every case and that the problem arose in environmental contamination of the sample, incorrect database matching and poor curation of proteins identified. In summary, this study exemplified that reproducibility in MS experiments can be achieved by carrying out the MS experiments with care and by upgrading existing databases for their curation [39, 40].

The link between interactome research and structural biology is made by native mass sepctrometry of large protein assemblies, an emerging, very promising technology. Native mass spectrometry techniques allow sensitive analyses of endogenously expressed protein complexes with high speed and selectivity [41, 42]. Importantly, native MS can provide vital information about the structure, topology and architecture of protein complexes. Protein complexes in native MS experiments are prevented from disassociating in the gaseous phase during electro-spray ionization (ESI). Additionally, nanoflow ES (nano-ES) is employed for improved resolution of the sample being studied thereby improving the sensitivity of native MS [40]. High-perfomance mass analyzers, such as orthogonal ESI-time of flight (TOF) instruments, can be used to accurately identify ions with a high mass-to-charge ratio, a prerequisite for analyzing large protein complexes with many subunits by native MS [42]. Tandem MS-MS methods, usually used in proteomics experiments to deduce the amino acid sequences of small peptides, can be applied to native MS to gather information about the subunits present in a protein complex [40]. Apparently, peripheral subunits are preferentially eliminated in this setup, thus allowing interpretation of the topology of the complexes analyzed.

A recent technological advance is ion mobility seperation coupled to mass spectrometry (IM-MS), which has been particulary useful to establish mass spectrometry as a powerful tool for structural biology applications [41, 43]. In IM-MS, ions are separated on the basis of their mass-to-charge ratio and as well on their drift time in a gas-filled ion mobility chamber. The drift time depends on the cross-section of the molecule, with larger molecules exhibiting longer drift-times, thus allowing determination of the average projection area of a specimen studied. It is conceivable that this technique will mature into a tool that will be routinely used to measure the cross-section of large protein complexes, which could be rather useful for providing volume constraints that can be utilized in molecular modelling of these assemblies [43].

Requiring relatively small amounts of protein sample compared to other MS techniques, nanoelectro-spray ionization can achieve the maintenance of a solution structure in the gas phase. Using collision-induced dissociation (CID), even very large protein complexes can be selectively dissociated by collision with neutral gas atoms. Each collision event results in the accumulation of internal energy by the ion in question. Upon accumulation of sufficient internal energy, this ion may undergo dissociation. This approach can be used to dissociate protein complexes into subcomplexes and subunits which are then analyzed with TOF instruments. CID has been used to analyze virus capsids and entire ribosomes with a molecular mass of 2.5 MDa [44]. The complete subunit architecture of the yeast exosome, the protein machine which degrades RNA in yeast, could be correctly assigned using CID [45]. Furthermore, subcomplexes and peripheral subunits of human elongation factor elF3 could be identified by using this method [46, 47].

## IMPACT OF STRUCTURAL GENOMICS

The description of the 3-D structure of biological macromolecules, at near-atomic resolution, is imperative for understanding their function at the molecular level. The elucidation of the DNA sequence of the entire genome of many organisms, including humans, revealed the gene repertoire present in cells. This set the stage to address the proteome, which is the comprehensive assemblage of all known gene products in an organism. The elucidation of the 3-D structure of all encoded proteins, at high resolution, is the goal of structural genomics efforts. Structural genomics aims at building up a high-resolution library dedicated to cataloguing the protein complement of different organisms *via* high-throughput and automated approaches starting from molecular cloning of the genes to structure elucidation of the encoded proteins. Based on structures deposited in the Protein Data Bank (PDB), structure determination by single crystal X-ray diffraction analysis is currently the predominantly used technique, in addition to structure determination in solution by NMR. By means of comparison with structures of well-characterized proteins and domains, the biological function of uncharacterized proteins can often be discovered or proposed. Until the beginning of 2008, the combined effort from structural genomics consortia worldwide contributed about 50% of the newly-deposited structures in the PDB. One of the largest structural genomic projects is the Project Structure Initiative (PSI) in the United States, which is sponsored by the National Institute of Health (NIH). Several other large consortia exist in Japan, Canada, and Europe [48].

In addition to the very large number of structures to be elucidated for describing a proteome, structural genomics approaches were confronted with a multitude of challenges. Successful structural determination by X-ray crystallography typically requires iterative optimization of protein encoding sequences for expression and purification of the specimens. Several to many expression vectors, host organisms and host strains need to be integrated into the experimental work-flow, in addition to covering a large space of conditions suitable for crystallization. All steps involved require considerable investment in labor and materials and a very significant through-put of experiments. Entire proteomes are addressed most often at the single protein or protein domain level. Consequently, structural genomics intensively stimulated and fostered the implementation of automation and high-throughput approaches, which now result also in considerable benefit for classical, hypothesis driven structural molecular biology. Many laboratories are now in the process of integrating high-throughput approaches at varying levels in their research [49].

Structural genomics projects generally start from target selection, which is based on evaluation of a large amount of candidate genes *via* bioinformatics methods. This is followed by cloning, insertion in one or several expression vectors, expression and purification, and finally structure determination. Researchers at centers engaged in structural genomics integrated automated cloning strategies based on restriction/ligation [50, 51], ligation-independent cloning [52, 53], or recombination [54, 55]. Among them, recombination based cloning systems are most widely utilized in high-throughput experiments. Although the systems used currently are robust and can be automated, they are often not sufficiently flexible when variations of expression elements such as purification tags, promoter/terminator combinations, protease cleavage sites and others need to be introduced or modified [49].

Autoinduction procedures were found to be particularly useful for automated high-throughput approaches for expression of the target specimens in *E. coli* as expression host. Autoinduction is based on a defined medium containing glycerol, glucose and lactose as inducer, which makes use of promoters containing *lac* operators. Glucose prevents induction by lactose until it is consumed. Upon glucose depletion in the culture, lactose is metabolized and heterologous induction occurs by means of the *lac* operator. Autoinduction thus simplifies the expression procedure: it alleviates the requirement for monitoring the density of cell cultures, as glucose depletion auto-regulates the time of induction. Further, auto-induction does not require the addition of inducer chemicals facilitating means for automation [56].

Increasingly, cell-free (CF) protein synthesis methods emerge as a viable alternative to *in vivo* expression in structural genomics pipelines due to several advantages [57]. Proteins that are toxic to host cells can be expressed by CF expression, and CF expression, in principle, can be better controlled by using highly purified components [58]. CF expression is especially useful for structure determination by NMR spectroscopy, since it is performed in small volumes and therefore requires less isotope label than cellular protein labeling [48, 57]. CF methods may be particularly useful for efficient screening of detergents required for successful production for membrane proteins [59], and may also allow rapid, small volume parallel screening of many variants of a target protein [60].

Many particularly exciting targets in the proteome will require expression in eukaryotic systems. Baculovirus expression vector systems (BEVS) increasingly become the method of choice for many of these targets. While considerable effort is being invested into automation and high-throughput protein expression by using BEVS [61-63], controlled virus generation in sufficient quantity and quality remains a challenge with currently available BEVS technologies [61]. Transient transfection of plasmid DNA into the nucleus of insect cells was suggested as a possible, economic alternative for analytical screening prior to larger scale virus generation [61].

Hierarchal multiplex expression and purification strategies utilized by the core Protein Production Platform of the Northeast Structural Genomics Consortium (NESG), foster an increase in the production of protein samples and also the solution of many 3-D protein structures [55]. Initiatives are ongoing to set up productive modules for target sampling, cloning, sample characterization and crystallization, arranged into fully integrated pipelines [64]. Since compact globular domains defined by limited proteolysis are good candidates for production of diffraction quality crystals, high-throughput limited proteolysis/mass spectrometry approaches for protein domain elucidation are being included into such pipelines, providing precise definition of domain boundaries, with significant impact for success prospects [65].

Structural genomics has decisively accelerated automation and the development of robust high-throughput methods. Nonetheless, critics claim that structural genomics consortia have gone after the “low-hanging fruit”, such as soluble single proteins of prokaryotic origin which are comparatively easy to express and purify [66]. Actually, structural genomics efforts now are gradually moving to address more challenging target proteins of eukaryotic origin. The objective is to facilitate the structural determination of human proteins, integral membrane proteins, and eventually multiprotein complexes [48]. However, the currently implemented approaches for automation and high-throughput methods cannot easily accommodate the upgrade required to address, in particular, large and complex multicomponent systems. The automation currently implemented in cloning routines and expression systems are mainly designed for addressing single ORFs or small, mostly binary systems [67].

## EUKARYOTIC MULTIPROTEIN EXPRESSION: MULTIBAC

The interactome can not be rationalized on the basis of elucidating single protein structures. It is now increasingly clear that the proteins in the cell function as interlocking machines containing ten or more interaction partners, that associate stably or transiently to realize cellular activities [1]. Structural genomics efforts have provided a wealth of detail on the level of individual proteins and domains. To address the more complex challenge of multicomponent assemblies, a number of expression systems have been introduced, that are suitable for simultaneous expression of several genes in prokaryotic and eukaryotic hosts [68-72]. In spite of considerable improvements of eukaryotic expression systems, *E. coli* still remains to date the expression system of choice in most laboratories. Nonetheless, eukaryotic expression is also being implemented for production of samples that can not be produced in *E. coli*. In particular the baculovirus/insect cell system has been streamlined significantly, and detailed protocols have become available that considerably simplify handling, thus alleviating some of the uncertainties regarding this system that impeded its routine application by non-specialist users [70, 73, 74]. 

Our laboratory has contributed to some of these developments, with particular focus on the production of multicomponent protein complexes for structural biology applications. We are interested in the structural molecular biology of eukaryotic complexes. For recombinant overproduction of these complexes, a system for multiprotein expression in insect cells, called MultiBac, was introduced [70, 73] (Fig. (**2**)). MultiBac uses an engineered deletion baculovirus with improved protein production properties including reduced proteolysis and a delayed onset of cell fragmentation in the late phase of viral infection [73]. This MultiBac baculovirus is accessed by two plasmids called transfer vectors at two recombination sites present on the virus: a LoxP imperfect inverted repeat for site-specific recombination, and a Tn7 attachment site. The Tn7 attachment site is embedded in a LacZα gene for blue-white selection of recombinant baculoviruses. These transfer vectors harbour the heterologous genes of interest. The MultiBac baculovirus exists as a BAC in *E. coli* cells containing also a small plasmid with four genes encoding for the Tn7 transposon, similar to the widely utilized Bac-to-Bac system from Invitrogen, and essentially all other baculovirus systems that rely on Tn7 transposition of a transfer vector *in vivo* in an *E. coli* host strain.

The transfer vectors that we developed for MultiBac contained elements that made it particularly straight forward to arrange into multigene expression cassettes several to many expression units containing ORFs encoding for example for members of a protein complex of choice. One transfer vector was designed to provide these multigene cassettes between Tn7L and Tn7R DNA sequences for integration into the Tn7 site of the MultiBac baculovirus. A second transfer vector contained a LoxP sequence thus enabling integration of multigene cassettes into the LoxP site of the MultiBac virus in the presence of *Cre* recombinase, the enzyme responsible for fusing DNA pieces that contain the imperfect inverted repeat. Integration into the LoxP and Tn7 site could be carried out simultaneously by co-transfecting the two transfer vectors into *E. coli* cells harboring the MultiBac virus, and expressing Tn7 transposon and *Cre* recombinase, respectively, from helper plasmids [73]. Selection for recombinant MultiBac viruses harboring the multigene cargo occurred *via* blue/white selection and antibiotic challenge for the resistance marker contained in the plasmid incorporated into the virus by* Cre*-LoxP fusion (Fig. (**2**)).

The MultiBac system as conceived in 2004 was surprisingly well received in the community, probably indicating the present and growing interest in researching eukaryotic interactomes and multiprotein complexes. Many laboratories requested the MultiBac reagents, many proteins were expressed, and X-ray crystal structures based on specimens produced by MultiBac are now being reported [75, 76]. Interestingly, our baculovirus expression technologies were not only used successfully for protein complex production for structural biology as they were designed for, but also for rather diverse other applications ranging from production of possible vaccine candidates based on papilloma virus like particles [77] to preparing recombinant adenoviruses for gene therapy treatment of obesity in laboratory rodents [78].

In our view, the genuinely useful contribution in conjunction with MultiBac, was not only the creation of yet another baculovirus and a few transfer vectors. We had realized in the process of our experimental work that the parameters of virus generation are not really compatible with routine application of an expression method in laboratories focusing on structural analysis. Baculovirus expression is constrained by certain requirements that need to be met to assure that the recombinant DNA cargo is properly maintained in the baculoviral genome during virus amplification and eventually protein production [79-81]. We found that introducing a fluorescent marker gene into the virus backbone, and precisely monitoring fluorescence intensity as well as the cell growth development in a culture, provided a very useful and simple regimen to largely alleviate the detrimental loss of titer or loss of protein production which are the major impediments encountered when using BEVS. This allowed us to establish a robust protocol for virus generation, amplification and protein production which then could be applied routinely and successfully in our laboratory and many others including non-specialist users [74]. We feel that BEVS expression, by using these protocols, can now be performed with almost the same ease and effort, as heterologous expression is commonly carried out in *E. coli*.

## ACEMBLING MULTIPROTEIN COMPLEXES

The combination of many genes encoding for subunits of a protein complex into vectors used for expression will remain a rather laborious task, in particular if it relies on restriction digestion and pasting together of DNA fragments by ligase in a serial, one-gene-at-a-time mode. This approach is essentially refractory to automation. Structural genomics consortia have strived to address the problem by implementing recombination methods for gene insertion. These methods have the advantage that they always use the very same reagents and reaction conditions, and therefore can be scripted into a robotics routine. The emphasis of most systems currently was mainly placed on offering a multitude of expression options for the one ORF of choice. For instance, the Gateway system from Invitrogen, defines an Entry vector for the gene of interest, which is inserted by any suitable means. This Entry vector is then used to introduce this gene into a wide range of Destination vectors providing a large assortment of purification or solubility tags for expression in a variety of hosts. The situation presents itself in reverse for multiprotein complex expression: here, the challenge is to introduce an assortment of genes into probably one expression system of choice to start with. This needs to be achieved in a way that ideally, the genes encoding for the multiprotein complex to be studied can not only be assembled fairly easily, but also options need to be provided to modify the individual subunit components rapidly and in a flexible way by mutation, truncation or replacing of affinity tags. Already for single proteins, altering the wild-type sequence for example by removing low complexity regions is often a prerequisite for successful high-resolution structural analysis, and introducing mutations is commonplace for elucidating the function and activity. This is equally valid for multiprotein complexes, however, the tasks at hand are considerably more complicated to achieve as the number of interacting subunits increases.

These deliberations and underlying experimental necessities prompted us recently to introduce ACEMBL, an automatable system for multiprotein expression making use of multigene recombineering by using a robot [82, 83] (Fig. (**3**)). For matters of simplicity, we first created ACEMBL in a version suitable for multiprotein complex production in *E. coli* as an expression host, although, the same robotic scripts can likewise be applied for generating multigene constructs for protein complex expression in eukaryotic hosts. We decided to consequently adapt recombination methods at every step of the process of gene insertion and gene combination into multigene expression cassettes, and to implement already existing, robust robotics protocols for small scale expression and protein extraction by using affinity purification [82].

Building on our positive experiences using *Cre*-LoxP fusion in MultiBac, we synthesized two families of small plasmids with the minimum DNA sequences required. These plasmids are called Acceptors and Donors. They are small (2-2.5 kb) and each plasmid contains the LoxP inverted imperfect repeat. Donors contain a conditional origin of replication which makes their existence and propagation in regular cloning and expression strains dependent on *Cre*-LoxP mediated fusion with Acceptors, which in turn have a regular origin of replication derived from the classical ColE1 origin.

We settled on sequence and ligation independent cloning (SLIC) as the method of choice for inserting genes into Donors and Acceptors, as detailed protocols for this methods became available recently [84]. Nonetheless, we needed to modify and improve these protocols to achieve robust integration, in particular when the process was carried out on in a robotic setup using a liquid handling workstation [82, 83]. This SLIC method, and likewise the BD-InFusion (Clontech Takara) or standardized ligation independent cloning (LIC) methods (Novagen), are commonly referred to as recombination methods, although this denotion is slightly misleading for these approaches. Rather, these methods have in common that they make use of the 3’ exonuclease activity of DNA polymerases in the absence of nucleotide triphosphates. Thus, long single stranded overhangs are created which can serve as sticky ends if complementary single strands become available. Nicks are closed and gaps are filled by the *E. coli* machinery upon transformation with the annealed DNAs. We found that efficient procedures could be established for integrating single genes or polycistrons into the ACEMBL Donors and Acceptors by SLIC, and scripted into robust routines, which could be readily carried out by a robot [82]. Gene integration into the ACEMBL vectors occurs at integration sites that make up a so-called multiple integration element (MIE), which contains also restriction sites for conventional gene integration as well as homing endonuclease sites for facile gene multiplication into multi-expression cassettes [82].

Donors thus charged with recombinant DNA cargo, each containing single genes, polycistrons or multiple expression cassettes, are then fused with one Acceptor by using *Cre* recombinase and the LoxP site present on each vector. Acceptors like Donors can contain one or several genes, polycistrons or a combination thereof. Several Donors can be fused with each Acceptor. Selection for multiple resistance, each of these characteristic for one Donor or one Acceptor, then identifies the Donor-Acceptor fusions in a combinatorial fashion. By using this approach, we could easily generate in a single reaction a series of multigene expression vectors expressing protein complexes as well as all possible combinations of genes contained on the individual vectors, revealing subcomplexes [82]. Interestingly, our experiments showed that multigene expression vectors could not only be assembled in this way, but likewise also selectively deconstructed by using the reverse approach. This is achieved by applying *Cre* recombinase to previously generated Donor-Acceptor fusions. This is possible due to the equilibrium reached between the fusion and excision activities of the *Cre* enzyme. Thus, defined parts of a multigene construct, encoding for subunits of a protein complex, can be excised by our procedure, altered for example by truncation, mutation, or replacement of the encoding genes, and then reintegrated into the multigene expression construct of choice by applying *Cre* fusion. This provides useful combinatorial options, also for robotics applications [82]. By employing the ACEMBL method, we were able to express and purify all members of the holotranslocon from *E. coli*, a large prokaryotic translocation complex consisting of six transmembrane proteins, from a 16 kb multigene plasmid [82].

## STRUCTURAL COMPLEXOMICS?

Genome and proteome-wide studies have clearly revealed the key role of macromolecular complexes in most, if not all vital cellular processes. Protein complexes display activities that are entirely different from the activities of each subunit studied independently, as interaction partners often dramatically influence recognition propensities and likewise biological activities. In addition, protein complex composition in particular in higher eukaryotes can depend on tissue type and cell state. Importantly, covalent posttranslational modifications such as phosphorylation, acetylation, methylation and many others can have a critical impact on the formation of protein complexes and their activity. Due to all of the variables that need to be controlled when attempting to assemble protein complexes recombinantly, it is important to have a robust system that allows rapid testing of many different constructs.

In the current environment, in which valuable information about interactomes, complexomes and other genome-wide studies is pouring in at an ever increasing pace, structural biology as it is performed to date simply cannot keep up with the increasing demand for the validation that only 3-D structures can provide. Protein structures can offer insights into the details of a protein interaction at the molecular or near-atomic level, and it is imperative for structural biologists to move into the arena of protein complex interactions. Despite recent colossal efforts in obtaining 3-D structures at near atomic resolution by X-ray crystallography, greatly fostered by structural genomics consortia, obtaining diffraction quality crystals of protein complexes remains a significant challenge and often takes on the order of years achieve. This technological state-of-the-art is simply incompatible with the speed at which new data is accumulated through high-throughput research addressing the interactome, and a major effort towards the development of new technologies is urgently required to close this gap.

3-D structural information can be gained from purified material extracted in small amounts from native source by electron-microscopic techniques which have significantly matured in recent years [85-87]. In particular, cryo-electron microscopy in conjunction with single-particle analysis can be used to gain information about the quaternary architecture of multiprotein assemblies. Although 3-D protein structures obtained from cryo-electron microscopy are reaching higher resolutions than ever before, 3-D structures obtained by this method provide still limited information when compared to the atomic details obtained by X-ray crystallography or NMR spectroscopy.

Undoubtedly, great benefit could be derived from the development of advanced techniques and reproducible protocols for micropurification of endogenous complexes. Purification of protein from biological material present in limited amounts will certainly be necessary in particular for the identification of complexes, or variations of complexes, that are present in specialized cells or specific tissues, and for a thorough validation of interactome data. This requires highly efficient methods to recover the quantities of protein required for biophysical methods. Due to the considerable increase in sensitivity of mass spectrometers achieved in recent years, it is now possible to routinely identify subunits of protein complexes from pico- to femto-mole quantities of material. It is critically important now to develop new strategies for the micropurification of protein complexes that will allow the simultaneous processing of several samples from limited amounts of source material. Such micropurification techniques, in conjunction with process automation for endogenous sample preparation will decisively improve current research approaches both in terms of throughput and also quality of analysis. Size-exclusion chromatography (SEC) is often a rate limiting step in the preparation of protein complexes. New purification strategies involving native gels, capillary electrophoresis or absorption onto membranes could possibly mature into genuine alternatives to SEC, thus allowing parallel processing of many samples and increasing sample homogeneity.

Recombinant expression most certainly had a decisive impact on life science research, and is to date the major technique for successful production of well-defined macromolecular specimens in the quality and quantity required for many applications. Apart from notable examples such as ribosomes or RNA polymerase [88-91], near-atomic structure determination of complex multicomponent systems will in all likelihood in most cases depend on recombinant overproduction. More recently, several multi-expression systems have been introduced for expression of protein complexes in a variety of different expression hosts, two of these were described in some detail in this contribution. However, most systems currently available still require dedicated expertise and considerable technical specialisation of the user, which is refractory to routine research, in particular for high-throughput applications. Biological and also pharmaceutical research often depend on introducing variations (mutation, truncations, fusions with markers, etc) into the specimen studied. Multi-expression systems therefore must provide the flexibility required for rapid revision of experiments, where such alterations can be introduced with ease. The ACEMBL system we developed could represent a first step in this direction. Nonetheless, production of many vital protein complexes, especially those requiring a eukaryotic host machinery for sample production, remains a challenge and a major bottleneck in the pipeline to high-resolution 3-D structures.

A further consideration in protein complex biology are those complexes that contain protein subunits as well as RNA components which may need to be co-expressed for proper complex assembly and folding. Protein-RNA complexes such as telomerase, snRNPs or RNAi containing complexes are a focus of contemporary research efforts aimed at elucidating mechanisms of health and disease. The recent 3-D structure of a human spliceosomal U1 snRNP compellingly demonstrates the power of recombinant reconstitution of such a complex for structure elucidation [92]. Technologies allowing routine multigene expression in prokaryotic and eukaryotic hosts will certainly need to incorporate the means for producing heterologous complexes containing non-protein components such as RNA and other biomolecules.

Automation is essential for accelerating contemporary protein science. Automation depends on standardization and simplification of protocols that are robust and reproducible. These requirements must be addressed by the development of easy-to-use, affordable reagents that are ideally compatible with robotic procedures. Automation has already had a considerable impact on cloning, DNA preparation, protein purification by affinity tags and assaying protein activities. Protocols optimized for automation have at times superseded earlier, more laborious procedures even in laboratories not applying robots routinely, as manual procedures generally also benefit considerably from the standardization and robustness inherently required for methods that can be used by robots. Automation will be particularly important for reconstitution of macromolecular complexes by heterologous multigene expression as probably a large number of constructs will need to be tested for many cases until a satisfactory reconstitution is achieved, yielding specimens suitable for detailed studies. The number of possible combinations increases dramatically with the number of subunits. This is particularly true if the pipeline is geared towards X-ray crystallography.

In single crystal structure determination by X-ray diffraction, a vital prerequisite is the ability of a specimen to arrange into a highly ordered crystal lattice that diffracts the incident X-ray radiation to near-atomic resolution. Often, this challenge can only be met by introducing variation into the wild-type sequence until a crystallizable specimen is obtained. Limited proteolysis, in conjunction with mass spectrometry, has been particularly useful for defining regions of low-complexity that can often interfere with crystallization. Such regions are then typically removed by introducing truncations or deletions in encoding DNA sequences, and recombinant overexpression of the resulting variant can then result in sample more amenable to crystallization. Corresponding procedures are now being introduced in more elaborate structural genomics pipelines. Nonetheless, it is clear that implementing such limited proteolysis procedures, often already laborious for single proteins, will be vastly more complicated when several to many ORFs need to be diversified concomitantly in a multiprotein complex. Recent advances in mass spectrometry, including quantitative, multiplexed techniques [93, 94] may prove to be invaluable for designing tools to analyze limited proteolysis experiments of complex multiprotein assemblies in high-throughput for structure elucidation.

High-resolution structure determination, in particular by X-ray crystallography, has developed into an indispensable technology which can be readily applied to elucidate molecular function in near-atomic detail. While the field of X-ray crystallography has achieved considerable advancements in recent decades, namely in the design of automated crystallization platforms, robotics and greater access to high-brilliance synchrotron radiation sources, there is still a considerable distance to be covered before X-ray crystallography can tackle the number of challenges presented by interactome wide studies and complexomics. Miniaturization and standardization are now indispensable components of high-throughput crystallization platforms. High-throughout methods will continue to provide many exciting possibilities for crystallization experiments aided by the arrival of technologies requiring unprecedented small amounts of sample for screening a very large space of crystallization conditions. Structural genomics consortia have played an indispensable role by installing automated pipelines for solving 3-D structures of individual proteins and protein domains. The discovery of a vast plethora of multicomponent assemblies that form the interactome, their modifications, overlaps and variations poses a challenge for similar efforts that may appear seemingly unmanageable at the moment. What is now required is a concerted effort to advance current technologies as well as to develop and implement new methods and procedures for addressing the complexome of organisms.

## Figures and Tables

**Fig. (1):**
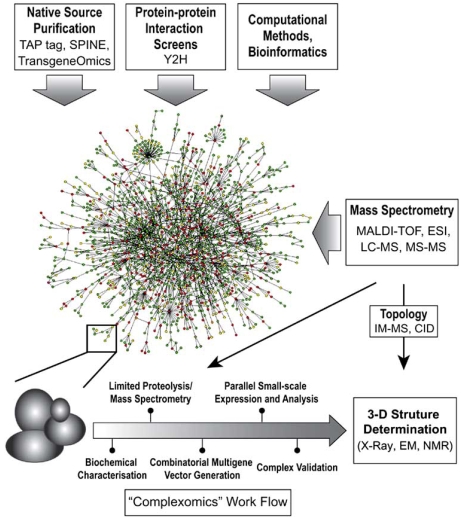
**Interactomics.** Recent technological advances in genome-wide methods enable researchers to address protein-protein interactions present in the proteome of organisms in a comprehensive fashion, thus giving rise to the interactome. Native purification of proteins present in organelles and entire cells by using tandem affinity purification (TAP) methods, Strep-protein interaction experiment (SPINE) and transgenomics involving bacterial artificial chromosomes for generating stable mammalian cell lines, as well as protein-protein screens by yeast two-hybrid (Y2H) methods are supported by bioinformatics analyses, and together provide a (growing) picture of the interactome as a complex mixture of multiprotein assemblies. Mass spectrometry (MS) based proteomic methods including matrix-assisted laser desorption ionization (MALDI) and electro-spray ionization (ESI) techniques coupled to liquid chromatography (LC-MS) and tandem MS-MS measurements add to the catalogue of tools employed to tackle the complexome. The link between ineractome research and structural biology is made by native mass spectrometry. Native MS can provide vital information about the structure, topology and architecture of protein complexes preserved in the gaseous phase. Ion mobility separation coupled to mass spectrometry (IM-MS) and collision induced dissociation (CID) are new approaches holding particular promise for characterizing the properties and composition of even very large protein complexes. Recombinant overproduction, functional characterization and eventually 3-D structure determination can help to validate the vast amounts of interactome data from recent systems biology efforts. Multiplexed and quantitative MS methods in conjunction with limited proteolysis may become critically important to elucidate variants of recombinantly overproduced multiprotein complexes amenable to high-resolution structural and functional analysis. Combinatorial multigene generation, parallel small-scale expression and biochemical and biophysical analysis of multiprotein complexes derived from interactome data constitute likely modules of a conceptual “complexomics“ pipeline in analogy to current structural genomics approaches, leading to routine and rapid elucidation of the molecular architecture of many complexes and their subunit components by X-ray diffraction analysis, electron microscopy and NMR spectroscopy.

**Fig. (2):**
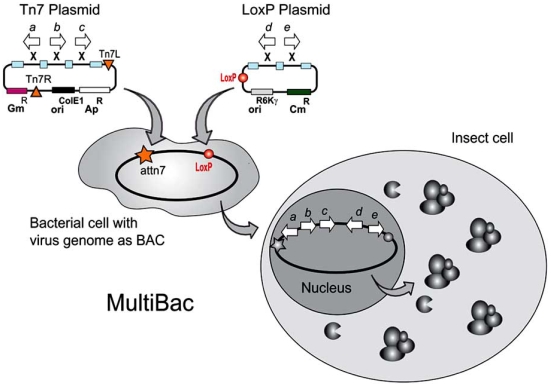
**MultiBac BEVS: Eukaryotic multiprotein expression.** ORFs (a-e) encoding for subunits of a protein complex and auxiliary protein such as modifiers or chaperones, are inserted into a plasmid containing the sequences required for Tn7 transposition (Tn7L, Tn7R), or a plasmid containing a LoxP imperfect inverted repeat, respectively. Gene insertion occurs *via* a multiplication module (small rectangles) designed for facilitating multigene cassette generation. A baculovirus genome containing the Tn7 attachment site (attn7) and a LoxP sequence, in addition to deletions beneficial for protein production, is present in bacterial cells in form of a bacterial artificial chromosome (BAC). Integration of multigene expression cassettes is mediated by the Tn7 transposon and *Cre* recombinase, respectively, which are expressed from helper vectors in the bacteria [73]. Transfection of insect cells with the resulting composite baculovirus results in high-level expression of the proteins in cultured insect cells. Adapted from [95].

**Fig. (3):**
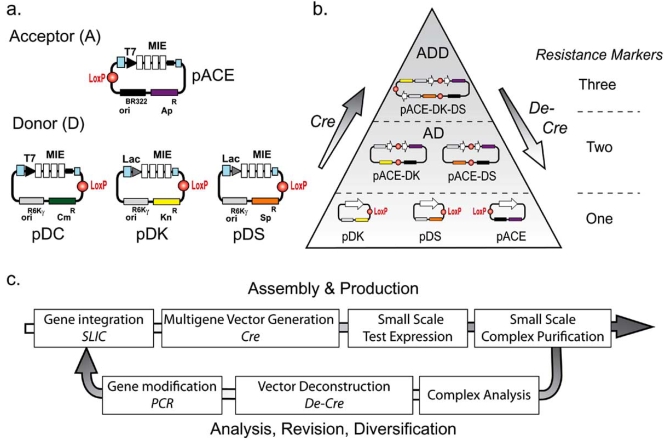
**ACEMBL System.** ACEMBL consists of newly designed, small vectors (**A**) and automated procedures and routines relying on recombineering for gene insertion and vector fusion (**B**). Multigene expression constructs are generated by insertion of genes into multiple integration elements (MIE) by recombination, followed by *Cre*-LoxP fusion of Donors with an Acceptor. Incubation of educt constructs (here pDK, pDS, pACE) containing genes of interest (white arrows) results in all possible combinations in a single reaction including Acceptor-Donor (AD) and Acceptor-Donor-Donor (ADD) fusions as shown here schematically. Creation of even four-plasmid ADDD constructs has also been completed successfully in our laboratory [82]. All co-existing constructs have characteristic antibiotic marker combinations and resistance levels (right). Donor vectors contain a conditional origin of replication derived from R6Kγ, and thus act as suicide vectors in cloning strains devoid of the *pir* gene unless fused to an Acceptor with a regular replicon. A second Acceptor, pACE2, is identical to pACE except for the encoded marker which confers resistance to tetracycline rather than ampicillin (not shown). Plasmid pACE2 can be used in conjunction with pACE derivatives for example to co-express auxiliary proteins such as chaperones or modifiers [82]. (**C**) Recombineering workflow by using the ACEMBL system is shown. Genes are integrated in Donors or Acceptors by ligation independent methods such as SLIC followed by combinatorial multigene vector generation using *Cre*-LoxP fusion. Expression and purification provide protein complex for analysis. Multigene vectors are deconstructed by using *Cre* excision activity (De-*Cre*). Encoded genes are modified by PCR and reintegrated into the workflow by recombination in an iterative cycle. The entire process is compatible with automation, and was successfully scripted into a robotic routine. Adapted in part from [82, 83].

## ABBREVIATIONS

BAC: = Bacterial artificial chromosome
BEVS: = Baculovirus expression vector system
CBP: = Calmodulin-binding peptide
CID: = Collision-induced dissociation
CF: = Cell-free
DBD: = DNA binding domain
EM: = Electron microscopy
ESI: = Electro-spray ionization
GFP: = Green fluorescent protein
HUPO: = Human Proteome Organization
IM-MS: = Ion mobility seperation coupled to mass spectrometry
kb: = Kilobase
kDa: = Kilodalton
LC-MS: = Liquid-chromatography coupled electro-spray ionization
LIC: = Ligation independent cloning
MALDI: = Matrix-assisted laser desorption/ionization
MIE: = Multiple integration element
MS: = Mass spectrometry
NMR: = Nuclear magnetic resonance
ORF: = Open reading frame
PCR: = Polymerase chain reaction
PDB: = Protein Data Bank
PPI: = Protein-protein interaction
SEC: = Size-exclusion chromatography
SLIC: = Sequence and ligation independent cloning
SPINE: = Strep-protein interaction experiment
TAP: = Tandem affinity purification
TOF: = Time of flight
Y2H: = Yeast two-hybrid
YFP: = Yellow fluorescent protein
